# The Urokinase Receptor: A Multifunctional Receptor in Cancer Cell Biology. Therapeutic Implications

**DOI:** 10.3390/ijms22084111

**Published:** 2021-04-16

**Authors:** Anna Li Santi, Filomena Napolitano, Nunzia Montuori, Pia Ragno

**Affiliations:** 1 Department of Chemistry and Biology, University of Salerno, Fisciano, 84084 Salerno, Italy; alisanti@unisa.it; 2 Department of Translational Medical Sciences, “Federico II” University, 80135 Naples, Italy; filomena-napolitano88@hotmail.it (F.N.); nmontuor@unina.it (N.M.)

**Keywords:** urokinase receptor, uPAR, cancer hallmarks

## Abstract

Proteolysis is a key event in several biological processes; proteolysis must be tightly controlled because its improper activation leads to dramatic consequences. Deregulation of proteolytic activity characterizes many pathological conditions, including cancer. The plasminogen activation (PA) system plays a key role in cancer; it includes the serine-protease urokinase-type plasminogen activator (uPA). uPA binds to a specific cellular receptor (uPAR), which concentrates proteolytic activity at the cell surface, thus supporting cell migration. However, a large body of evidence clearly showed uPAR involvement in the biology of cancer cell independently of the proteolytic activity of its ligand. In this review we will first describe this multifunctional molecule and then we will discuss how uPAR can sustain most of cancer hallmarks, which represent the biological capabilities acquired during the multistep cancer development. Finally, we will illustrate the main data available in the literature on uPAR as a cancer biomarker and a molecular target in anti-cancer therapy.

## 1. Introduction

Proteolysis is a fundamental event in several biological processes; at present, more than 550 human proteases are actually known, representing the second most abundant class of enzymes after ubiquitin ligases [1]. Proteolysis must be temporally and spatially regulated, its improper activation leading to dramatic consequences. Proteolysis regulation is realized through multiple mechanisms; they include not only the transcriptional and post-transcriptional regulation of the expression of proteases and of their inhibitors, but also the organization in cascades of sequential proteolytic activations of intermediate molecules, leading to final, biologically relevant, products. Fortelny et al. mathematically modelled protease interactions; their model includes 1230 proteins and shows connections between 141,523 pairs of proteases, substrates, and inhibitors [2].

Deregulation of proteolytic activity characterizes many pathological conditions, including cancer. In fact, extracellular proteases can regulate bioavailability of growth and pro-angiogenic factors, activity of other proteases, cell–cell and cell–matrix interactions. The possibility to concentrate proteolytic activity on the cell surface represents another layer of regulation of proteolytic activity, particularly in cell migration, allowing the cell to cleave the surrounding extracellular matrix (ECM) and migrate through it [3].

Among the proteolytic systems involved in cancer is the plasminogen activation (PA) system, which includes serine proteases as plasmin and the urokinase-type (uPA) and tissue-type (tPA) plasminogen activators, specific inhibitors, cellular receptors. uPA binds to a high-affinity specific cellular receptor (uPAR); cell-bound uPA is able to cleave and activate plasminogen, which also can bind the cell surface through low-affinity receptors. Active plasmin, derived from plasminogen cleavage, is a broad-spectrum proteolytic enzyme, having as substrates, among others, components of ECM and pro-metalloproteases. uPA and plasmin binding to the cell surface concentrates and strongly amplifies pericellular proteolytic activity, thus leading to the ECM degradation required for an efficient cell migration [4]. In fact, PA enzymatic cascade occurs in physiologic and pathologic events that include cell migration, as, for instance, leukocyte recruitment in inflammation, tissue remodeling, wound healing, tumor invasion and metastasis. Further, active plasmin is involved in the regulation of activity of various important cytokines as IL-1, IL-6, and TGF-beta, since it is required for their release and activation [5]. 

The identification of the cell-surface receptor for uPA confirmed the importance of concentrating proteolytic activity pericellularly and the key role of uPA-uPAR in cell migration. However, in the following years, a large body of evidence clearly showed uPAR involvement in biological processes independently of the proteolytic activity of its ligand. In 1993, it was reported that the aminoterminal fragment of uPA (ATF), able to bind uPAR but lacking any proteolytic activity, could induce cell migration by activating intracellular signaling pathways [6]. This observation was even more surprising because uPAR lacks transmembrane and cytosolic domains, being anchored to the cell surface through a glycosylphosphatidylinositol (GPI) tail; therefore, it was not expected to be able to transduce signals inside the cell. Over the years, uPAR has been shown able to activate intracellular signals regulating various biological processes. 

In this review we will first describe this multifunctional molecule and then we will discuss how uPAR can sustain most of the activities which represent cancer hallmarks. Cancer hallmarks include biological capabilities acquired during the multistep cancer development: invasion and metastasis, angiogenesis, deregulated proliferation and survival, replicative immortality, reprogramming of energy metabolism, inflammation, and evading immune response. All these hallmarks are sustained by genome instability, which is itself the main cancer hallmark [7]. Finally, we will illustrate the main data available in the literature on uPAR as a cancer biomarker and a molecular target in anti-cancer therapy.

## 2. uPAR Structure, Interactors and Expression

### 2.1. uPAR Structure

uPAR is a heavy glycosylated protein consisting of three homologous domains (DI, DII, DIII) belonging to the Ly6/uPAR/a-neurotoxin protein domain family, characterized by a distinct disulfide bridge pattern that creates the three-finger Ly6/uPAR (LU) domain [8]. The structure of uPAR complexed with a synthetic antagonist peptide or ATF has been solved by X-ray crystallography. These structures showed that ATF is buried deeply within a large hydrophobic cavity defined by the three uPAR LU domains, while the large outer surface remains available for potential additional ligands [9,10]. Despite several efforts, the structure of the unoccupied human receptor has not been determined, whereas, recently, the structure of unoccupied murine uPAR has been determined [10,11].

uPAR was firstly identified in 1985, but only after five years the isolation of the purified protein and the sequencing of its cDNA were reported [12]. Indeed, uPAR is synthesized as a 313 amino acid residues polypeptide; post-translational carboxyl-terminal processing leads to the loss of last 30 residues and to the attachment of a GPI tail, that anchors the receptor to the cell surface (GPI-uPAR) [13]. The GPI-anchor allows uPAR to move along the cell membrane and to associate with lipid rafts. Lipid rafts are heterogeneous, dynamic, cholesterol- and sphingolipid-enriched membrane domains, which may function as active signaling platforms, concentrating signaling mediators inside the cell and signaling receptors outside [14]. The GPI-anchoring also implies that cell-surface uPAR expression can be regulated by phospholipases, which, hydrolyzing the GPI tail, may induce the receptor release. Indeed, uPAR release from the cell surface by GPI-specific phospholipases C or D has been demonstrated [15,16], whereas proteases involved in the juxtamembrane proteolytic cleavage of uPAR have not been identified, even if they cannot be excluded. A shorter suPAR isoform can be also generated by alternative splicing of exon 7 in the DIII domain, leading to loss of the GPI anchor region [17].

The three uPAR domains are connected by two flexible hinges. The recent determination of the structure of unoccupied murine uPAR showed that DII and DIII form a compact globular unit; molecular dynamic simulations further confirm the rigid binding interface between DII and DIII [11]. 

The DI-DII linker region is instead particularly exposed and sensitive to the activity of several proteases, including trypsin, chymotrypsin, elastase, cathepsin G, metalloproteases, plasmin and uPA itself [18]. Interestingly, uPA is able to cleave only bound uPAR, in fact, inactivated uPA, after binding to uPAR, protects the receptor from the action of active unbound uPA [19]. 

The cleavage in the DI-DII linker region causes DI release and the generation of truncated forms of GPI-uPAR on the cell surface (DIIDIII-uPAR), exposing different N-terminus, depending on the cleaving enzyme and its consensus cleavage sequence. The expression of such cleaved forms of uPAR has been reported in both normal and tumor cell types [18]. 

Intact and cleaved uPAR forms have been detected also in soluble form in human fluids, both in vitro and in vivo [18]. 

### 2.2. uPAR Extracellular Ligands

uPA is secreted as the zymogen pro-uPA, consisting of a growth factor-like domain (GFD, residues 1–49), a Kringle domain (residues 50–131), an interdomain linker or “connecting peptide” (residues 132–158), and the serine protease domain (residues 159–411). Pro-uPA is activated by a proteolytic cleavage at Lys^158^-Ile^159^, which generates the two-chain active molecule; a further proteolytic cleavage can release the amino-terminal fragment (ATF, residues 1–135) and a small region linked to the large C-terminal proteolytic domain [20]. The GFD domain of uPA or ATF binds uPAR amino acid residues located mainly in the DI domain, even if secondary binding sites are present along the whole molecule; accordingly, full-length uPAR is required for an efficient binding to uPA [20].

Many efforts have been made to identify a cellular receptor for uPA, since it was expected that focusing uPA activity on the cell surface could amplify the plasminogen activation system cascade. The finding that the identified uPA receptor was a GPI protein further strengthened the idea that its sole and critical function was limited to the concentration of uPA proteolytic activity on the cell surface, given the lack of transmembrane/cytosolic domains able to transduce signals inside the cell. By contrast, uPA is able to regulate cell adhesion, migration, proliferation, and survival also independently of its enzymatic activity (12).

Further, over the years, the identification of ligands other than uPA clearly demonstrated the versatility of uPAR. In fact, unexpectedly, uPAR capability to bind vitronectin (VN), a component of provisional ECM, was demonstrated [21]. Five uPAR residues were identified as "hot spots" for VN binding; they form a composite epitope located at the interface between uPAR DI and DII and include the amino acid residues Trp(32), Arg(58), and Ile(63) of DI domain, and Arg(91) and Tyr(92) of the flexible linker peptide connecting uPAR domains I and II [22]. Also in this case, even if uPAR binding to VN involves amino acid residues in DI and DI-DII linker region, an efficient binding to VN requires full-length uPAR [23].

uPAR binds to the amino-terminal somatomedin B domain (SMB) of VN, in an RGD-independent manner. uPAR competes with the type 1 inhibitor of plasminogen activators (PAI1) for binding to VN, since PAI1 also binds the SMB domain of VN [24]. Thus, PAI1 inhibits both proteolysis and cell adhesion. 

By contrast, uPA increases uPAR binding to VN, possibly by controlling uPAR oligomerization and localization in lipid rafts [25]; however, biochemical analyses have shown a 1/1/1 stoichiometry of uPA/uPAR/SMB complexes, suggesting the need of alternative mechanisms, such as a uPA-driven allosteric regulation of uPAR [10]. 

These findings indicate that uPAR can form a ternary complex with uPA and VN, thus coordinating cell adhesion and pericellular proteolysis. The structure of the ternary complex, formed by uPAR, ATF, and the SMB-domain of VN has also been solved by X-ray crystallography [26].

### 2.3. uPAR Cellular Ligands

The finding that uPAR is a VN receptor and can mediate cell adhesion to VN implied that uPAR is able to transduce signals, despite being a GPI protein. Indeed, other previous evidence already suggested uPAR capability to activate intracellular signaling. In fact, inactivated uPA or ATF, upon binding uPAR, stimulated cell migration [6,27,28]. This uPAR activity needed cell surface signaling partners. Indeed, in 1994, Bouhslav et al. demonstrated that uPAR was a component of a large complex which included protein tyrosine-kinases and beta2 integrins expressed on the monocyte surface [29]. Since then, uPAR association with integrins, G protein-coupled receptors, receptor tyrosine-kinases (RTK) as epidermal growth factor receptor (EGFR), platelet-derived growth factor receptor (PDGFR) and insulin-like growth factor 1 receptor (IGFR) has been shown, indicating uPAR as a central player in an extensive interactome including over 42 interacting proteins [30]; among them, uPAR interaction with integrins, G protein-coupled receptors and EGFR are definitely the most investigated and characterized.

uPAR association with various families of integrins has been demonstrated by fluorescence resonance energy transfer (FRET) analysis, immunolocalization, co-immunoprecipitation [12], and binding between soluble uPAR, immobilized on a nitrocellulose membrane, and the purified alpha5beta1 integrin [31]. The first report suggesting uPAR association with a beta2 integrin showed co-capping of uPAR and the beta2 integrin Mac-1 in resting neutrophils [32]; uPAR co-immunoprecipitation with beta2 integrins and signaling molecules demonstrated that uPAR, despite the GPI tail, can be connected to intracellular signaling mediators [29]. In fact, it was later shown that uPAR ectopic expression in uPAR-negative HEK-293 cells allowed formation of stable uPAR-beta1 integrins complexes, affecting integrin adhesive function to fibronectin (FN) and promoting cell adhesion to VN. Both uPAR-mediated adhesion and altered integrin function were blocked by a peptide, P25, identified by a phage-display library, which bound to uPAR and disrupted uPAR-integrin complex [33]. P25 was then demonstrated to be highly homologous to a region between the ligand-binding I-domain and highly conserved divalent cation repeats of CD11b, the *a*(M) subunit of Mac-1, corresponding to amino acids 424–440 (peptide M25). M25 competes with beta2 and beta1 integrins for the binding to uPAR, impairing their association and regulating their activity [34].

Since then, uPAR interactions with integrins and the consequences on integrin activity have been largely investigated [12]. uPAR can bind integrins through amino acid residues located in the DII domain (residues 130–142, peptide D2A) [35] and in the DIII domain (residues 240–248, peptide 240–248) [31]. Peptides spanning these two regions abolish uPAR-integrins co-immunoprecipitation; substituting a single amino acid (S245A) in the DIII 240–248 peptide or in the full-length soluble uPAR impairs binding of the purified integrin (31); mutating two glutamic acids into two alanines in the peptide D2A inhibits VN-, FN-, and collagen-dependent cell migration [35]. Interestingly, D2A is endowed with chemotactic activity and promotes cell growth [35,36].

uPAR binding to integrins requires the full-length receptor [37], as well as uPAR-uPA and uPAR-VN interactions.

The importance of uPAR interactions with integrins has been documented also in vivo, showing that the P25 peptide significantly reduces tumor metastasis of MDA-MB-231 cells in bone [38].

A very intricate interplay between uPAR, integrins, and RTKs, in particular the EGFR, has been also reported. In fact, in HEp3 human carcinoma cells, uPAR can influence phosphorylation and signaling activity of the EGFR, independently of EGFR ligands and the level of EGFR expression. Highly expressed uPAR promotes EGFR interaction with the integrin alpha5beta1, leading, in the presence of FN, to the formation of a multiprotein complex including uPAR, EGFR, alpha5beta1, and the focal adhesion kinase (FAK), that causes robust ERK activation. Inhibition of EGFR-kinase impairs uPAR-induced signal to ERK, indicating that EGFR may act as a transducer of uPAR signal [39].

Important uPAR cell-surface partners are also the G protein coupled-receptors for the chemotactic formylated peptide fMLF (fMet-Leu-Phe). Formylated peptides are actively produced by invading pathogens or passively released from dead and dying host cells after tissue injury. Currently, three fMLF receptors (FPRs) have been identified and cloned, the high-affinity *N*-formyl-peptide receptor (FPR1) and its homologues FPR-like 1 (FPR2) and FPR-like2 (FPR3). FPR1 binds fMLF with high affinity, FPR2 has a much lower affinity for fMLF but can bind several other molecules, including lipoxin A_4_, serum amyloid A, HIV derived peptides. FPR3 shows a high homology with the other two receptors but it does not bind fMLF at all and shares few ligands with the other FPRs. FPRs were initially identified in neutrophils and monocytes/macrophages, in which they mediate chemotaxis; afterwards, FPRs expression has been demonstrated in other leukocytes and also in a variety of non-immune cells, including endothelial cells, synovial fibroblasts, keratinocytes, intestinal epithelial cells, bone marrow-derived mesenchymal, and hematopoietic stem cells, hepatocytes, suggesting their involvement in several and different biological activities [40]. FPRs are also expressed in various cancers and mediate motility and growth. For instance, in human gastric cancer cells, FPRs may mediate epithelial–mesenchymal transition (EMT), cell proliferation, migration, and survival. FPR1, in malignant glioblastoma multiforme (GBM) cells, is activated by the endogenous chemotactic ligand annexin 1 (ANXA1) released by necrotic GBM cells and functionally interacts with the EGFR to promote survival and invasiveness of GBM cells. Further, FPRs contribute to enhanced proliferation of human breast and colon cancer cells and to invasion, proliferation and production of angiogenic factors in human liver cancer cells [40,41]. 

The first evidence of a connection between uPAR activities and FPRs goes back to a report showing that uPAR expression was required for fMLF-induced monocyte chemotaxis in vitro [42]. Some years later, Blasi’s group showed that uPA-induced cell migration required FPR2 expression and demonstrated the direct binding between FPR2 and the cleaved form of soluble uPAR (DIIDIII-suPAR) containing the SRSRY sequence at its N-terminus. DIIDIII-suPAR was able to activate FPR2 and to induce chemotaxis of monocyte-like cells and monocytes [43,44]. Interestingly, while in soluble uPAR the SRSRY sequence has to be unmasked by DI removal in order to bind and activate FPRs, in the cell-anchored receptor the same sequence is exposed also in the full-length uPAR, acting as an endogenous FPRs ligand [45]; this finding was consistent with the observation that the hydrolysis of the GPI tail induces conformational changes of uPAR [46]. Indeed, we later showed that full-length uPAR co-immunoprecipitated with all three FPRs [47]. Moreover, uPAR, whose expression can be regulated by uPA both at transcriptional and post-transcriptional level [48], can in turn regulate uPA expression through interaction with integrins and FPRs [49].

Thus, the various uPAR forms appear to be functionally different; in fact, GPI- and soluble full-length uPARs bind uPA and VN and associate with integrins, whereas cleaved uPARs do not; by contrast, all uPAR forms, except the full-length soluble form, are able to bind FPRs. 

Indeed, we demonstrated that uPAR is able to recruit a large fraction of both FPR1 and beta1 integrins at the cell surface of HEK-293 cells, strongly promoting their colocalization when cells were stimulated with serum. We proposed that uPAR could act as a docking cell-surface molecule for both FPRs and integrins, recruiting and bridging them on the cell surface, in particular in lipid rafts, where uPAR is located because of its GPI-tail [47,50]. Lipid rafts play important roles in many pathophysiologic processes, including cancer. In fact, lipid rafts appear involved in the regulation of signal transduction in cancer, acting as scaffolds to enhance intracellular signaling cascades, playing a relevant role in tumor angiogenesis, cell adhesion and migration, EMT, and apoptosis [14].

### 2.4. uPAR Expression

uPAR expression can be regulated both at transcriptional and post-transcriptional levels [48]. Transcription factors, including AP1, PEA3/Ets, SP1, AP2, and the hypoxia-induced factor 1α (HIF1α) mediate uPAR transcription in cancer cells [51]. RNA binding proteins as HuR and hnRNPC bind the AU-rich element (ARE) in the 3’-untranslated region (3’-UTR) of the uPAR mRNA promoting its stabilization, whereas the tumor suppressor protein p53 promotes its degradation [51]. 3’-UTR of uPAR mRNA is also targeted by oncosuppressor microRNAs as miR-146a and miR-335, in acute myeloid leukemia [52].

uPAR expression in healthy tissues is limited, but it increases in processes including cell migration and tissue-remodeling, for instance during embryo implantation and placental development or epidermal wound healing. Increased uPAR expression may be observed in activated endothelium, smooth muscle cells and immune system cells, main players of inflammatory and immune responses [53]. Accordingly, up-regulation of uPAR expression is observed in the kidney during chronic proteinuric disease, in the central nervous system following ischemia or in neurodegenerative disorders such as Alzheimer’s disease and Creutzfeldt-Jakob disease, in atherosclerosis, in auto-immune diseases such as rheumatoid arthritis and systemic sclerosis, and in inflammatory bowel diseases consisting of chronic relapsing inflammatory disorders of the intestinal tract [30,54]. 

Cell migration is a central process in cancer invasion and metastasis formation; in fact, uPAR is highly expressed by various cancer cells and by non-malignant cells that infiltrate cancers [53,55]; up-regulation of suPAR levels has been also observed in plasma and serum from patients affected by various diseases, including hematologic malignancies and carcinomas [56]. High uPAR expression on cancer cells and/or elevated levels of suPAR correlate with poor prognosis; in some cases, for instance in acute myeloid leukemia, multiple myeloma, breast and ovary cancer, elevated uPAR level is associated also with resistance to chemotherapy, and, in papillary thyroid carcinoma, with reduced patient disease-free interval [57]. 

uPAR increase in cancer and its correlation with a poor prognosis is related to its involvement in most cancer hallmarks.

## 3. uPAR and Cancer Hallmarks

### 3.1. uPAR in Invasion and Metastasis 

Tissue invasion and metastasis formation is a very complex mechanism including several critical steps: cancer cells must detach from primary tumor mass, migrate and invade surrounding tissues, intravasate into the bloodstream, and extravasate and colonize a distant organ. The crossing of local and surrounding tissues and of basement membrane requires localized proteolysis, thus uPAR, able to regulate pericellular proteolysis, plays a key role in these events. However, the finding that uPAR is also able to activate intracellular signals strongly suggested that uPAR contribution cannot be limited to this activity. Indeed, uPA, whose level is strongly increased in various cancer tissues, upon binding uPAR, can stimulate cell migration independently of its proteolytic activity; uPA-induced cell migration requires the expression of FPRs, whose expression is also increased in various tumor types [41].

The soluble form of DIIDIII-uPAR, increased in several cancers and associated to poor prognosis, if exposing the SRSRY sequence at its N-terminus, can bind and activate all three FPRs, inducing cell migration both in vitro and in vivo [44,45,58]. 

The complex interplay between uPAR and FPRs influences also the activity of CXCR4, the SDF1 chemokine receptor [59]; CXCR4 is strongly up-regulated in various malignancies and can drive disseminating cells to metastasis sites [60]. Indeed, we demonstrated that the influence of uPAR expression on cell migration is even much broader. In fact, we have shown that inhibition of uPAR interactions with integrins and FPRs impairs migration toward serum of PC3 prostate carcinoma cells and of uPAR-transfected HEK-293 (uPAR-293) cells, although latter cells are perfectly able to migrate when lacking uPAR expression. Recently, we showed that removal or substitution of the uPAR GPI tail impairs uPAR capability to control cell migration, indicating a key role of the GPI tail in this uPAR function [47,50]. 

uPAR binding to uPA, which promotes pericellular proteolysis, is a central step in mesenchymal motility. However, uPAR expression is required also for the amoeboid motility of prostate and melanoma cancer cells occurring in a protease inhibitors-rich milieu [61].

A link between tumor progression and EMT, the epithelial–mesenchymal transition, was first observed in epithelial cancer cell lines: down-regulation of E-cadherin expression or function promoted invasion and fibroblast-like morphology in breast, lung, bladder, and pancreas epithelial tumor cell lines. Subsequently, TGF-β was identified as a major EMT-inducing cytokine. Currently, EMT has been well characterized, it includes loss of adherent junctions and apical-basal polarity and acquisition of a mesenchymal phenotype with motility and invasion capabilities. Various stimuli may up-regulate EMT-inducing transcription factors orchestrating morphological, cellular, and molecular changes [62]. Various reports suggest that the uPA/uPAR complex can stimulate EMT to promote cancer progression. Hypoxia, that frequently occurs during cancer progression, induces EMT and increases uPAR expression; uPAR silencing in breast cancer, medulloblastoma and nasopharyngeal carcinoma cells inhibits hypoxia-induced EMT, whereas uPAR over-expression mimics EMT in normoxia [63,64,65]. Further, uPAR-dependent cell signaling induces stem cell-like properties in breast cancer cells [66]. The transcriptional factor FOXM1c contributes to tumor EMT and metastasis by enhancing uPAR gene transcription in pancreatic cancer [67].

TGF-β plays a key role in EMT induction. A tight connection between the uPA/uPAR system and TGF-β has also been observed: TGF-β up-regulates the expression of both uPA and uPAR; uPA binds uPAR and activates plasminogen, which, in turn, can activate latent TGF-β, participating to a positive loop contributing to the development of EMT in cancer cells [68,69].

Altogether, most reports show an active role for uPAR in EMT, even though a recent report shows that, instead, uPAR expression is essential for maintaining the epithelial phenotype in neuroblastoma Neuro2a cells, since uPAR silencing induces the down-regulation of epithelial markers (E-cadherin, occludin, and claudin-5) and the increase of mesenchymal markers (N-cadherin, α-smooth muscle actin, and interleukin-6) [70]. 

### 3.2. uPAR and Angiogenesis in Cancer 

Formation of new blood vessels is a crucial step in cancer progression. In fact, new vessels allow blood to reach all districts of the growing tumor mass, providing nutrients and oxygen; further, new vessels allow invading tumor cells to reach distant sites to colonize. Indeed, new blood vessels can sprout from pre-existing vessels (angiogenesis) or can form by endothelial progenitor cells (EPCs) (vasculogenesis); however, EPCs can also contribute to angiogenesis [71]. During angiogenesis, endothelial cells (ECs) degrade basement membrane, migrate through the ECM, proliferate and organize in the new vessels, which can include locally recruited EPCs. In fact, ECs can produce the SDF-1 chemokine at the site of injury and locally recruit EPCs, which express CXCR4, the SDF1 receptor [72]. Interestingly, the soluble DIIDIII-uPAR can regulate CXCR4 activity through a mechanism involving FPRs [73]. 

The obvious contribute of uPAR to angiogenesis corresponds to its traditional role of concentrating uPA activity on ECs, thus promoting plasminogen and MMPs activation, leading to the localized ECM degradation required for ECs migration, and to the activation of angiogenic factors [74]. However, uPAR involvement, also in angiogenesis, goes further the uPA proteolytic activity. In fact, inactive uPA induces neovascular growth in the avascular rabbit cornea and promotes growth, chemotaxis and matrix invasion of cultured endothelial cells [74]. A pro-angiogenic signaling pathway, activated by inactive uPA and mediated by uPAR DII, beta1-integrins, and VEGF receptor 2 (VEGFR2), has been reported [75]. In HUVECs (human umbilical vein endothelial cells), uPAR promotes internalization of the complex VEGF-VEGFR2, by associating with VEGFR2 and recruiting the low-density lipoprotein receptor-related protein 1 (LRP-1). LRP-1 induces VEGF-VEGFR2 internalization, which activates multiple signaling pathways necessary for angiogenesis [76].

In endothelial cells, uPAR, through a mechanism involving integrins, is also able to down-regulate the expression of the tumor suppressor PTEN (phosphatase and tensin homologue), which negatively regulates angiogenesis [77]. 

Lipid raft localization of uPAR appears crucial also in angiogenesis. EPCs with a high proliferative rate (ECFCs) have been identified in human umbilical blood; VEGF up-regulates ECFCs expression of caveolin-1 and uPAR as well as their association with lipid rafts. An anti-uPAR antibody or uPAR silencing impair caveolae formation, ECFCs invasion and capillary morphogenesis [78]. Further, uPAR recruitment in caveolar-lipid rafts by GM1 ganglioside regulates EPC-dependent angiogenesis [79]. 

The cleaved form of soluble uPAR also shows angiogenic activities, promoting the formation of cord-like structures in vitro, through a mechanism involving FPRs, thus leading to sprouting in human saphenous vein rings and to a marked response in angioreactors implanted into the dorsal flank of nude mice [80]. 

uPAR is also expressed in melanoma cell lines-derived exosomes, which are internalized by HMVECs and ECFCs, enhancing VE-Cadherin, EGFR, and uPAR expression in endothelial cells that undergo a complete angiogenic program, including proliferation, migration and tube formation. uPAR loss reduces the pro-angiogenic effects of melanoma exosomes in vitro and in vivo [81].

### 3.3. uPAR and Replicative Immortality

Senescence has been initially associated with the cell-cycle arrest that occurs after cells have undergone a defined number of divisions in vitro; thus, senescence was considered as a brake to excessive proliferation. The limited growth of human cells in culture is due in part to telomere erosion, which occurs at each S phase of cell cycle; eroded telomeres generate a persistent DNA damage response which supports the growth arrest. Therefore, cellular senescence was thought to be a tumor-suppressor mechanism, impairing DNA-damaged potential tumor cells to proliferate. Telomerase, a ribonucleoprotein enzyme which is required for complete replication of the DNA ends, is not expressed by most human somatic cells, whereas it is expressed by most human malignant tumors, which can thus overcome the cell-cycle block [82]. 

Indeed, DNA double strand breaks, even at non-telomeric sites, are potent senescence inducers. Cellular senescence can also be induced by stimuli different from DNA damage, for instance by strong mitogenic signals, including those due to some oncoproteins, oxidative stress or loss of the PTEN tumor suppressor, a phosphatase that impairs pro-proliferative/pro-survival kinases [83]. Senescent cells acquire a senescent-associated secretory phenotype (SASP), which is characterized by a strong increase in the secretion of pro-inflammatory cytokines, which can promote degenerative or hyperproliferative changes in neighboring cells. The aberrant accumulation of senescent cells generates an inflammatory microenvironment leading to chronic tissue damage, which can contribute to various diseases, including cancer [84].

uPAR involvement in cellular senescence appears controversial. In fact, uPAR silencing in human vascular smooth muscle cells (VSMCs) results in abrogation of doxorubicin-induced cellular senescence, whereas uPAR overexpression promotes VSMC senescence, regulating the proteasomal degradation of telomeric repeat binding factor 2 (TRF2), which plays a key role in the protective activity of telomeres [85] and activates the DNA single-strand break repair signaling pathway [86]. By contrast, uPAR silencing induces senescence-associated nuclear morphology and induction of beta-galactosidase activity in papillary thyroid carcinoma cells [87]. 

Interestingly, suPAR is secreted by senescent cells as part of the SASP [88] and, recently, uPAR expression on the surface of senescent cells in vitro and in vivo has been reported and even targeted by senolytic CAR T cells [84]. 

### 3.4. uPAR in Cell Proliferation and Survival

The capability of uPAR-bound uPA to stimulate cell proliferation was observed many years ago [89], even though this effect was initially only attributed to focused uPA proteolytic activity, since plasmin and uPA can activate the transforming growth factor-β [5] and the hepatocyte growth factor [90], respectively. Indeed, the ability of ATF, lacking proteolytic activity, to stimulate cell proliferation demonstrated that the mechanism underlying the proliferative effect of uPA-uPAR could also be independent of proteolysis. Integrins, which can regulate cell proliferation, play a key role in this uPAR activity. In fact, association of overexpressed uPAR and beta1 integrins regulates two opposite pathways, activating ERK and inhibiting p38MAPKs, thus stimulating in vivo growth of carcinoma cells [91]; EGFR, which can associate with uPAR on the cell membrane, mediates the uPAR/integrin/fibronectin induced growth pathway [39]. Accordingly, primary uPAR-/- keratinocytes do not proliferate in response to EGF in vitro [92]. A mitogenic sequence of uPAR corresponds to the D2A uPAR fragment [35]; in fact, D2A synthetic peptide transactivates EGFR and is as potent as EGF in stimulating cell growth [36]. The same uPAR-derived peptide had been previously shown to stimulate cell migration [35]. By contrast, uPAR -/- mouse embryonic fibroblasts (MEFs) grow faster than wt MEFs and infection with a uPAR-containing retrovirus decreases their growth rate; proliferation of wt MEFs appears do not involve FPRs or integrins [93], which instead may contribute to the proliferative advantage observed in the other reports [91,92], suggesting different regulatory mechanisms in MEFs. 

uPA promotes uPAR association also with the PDGFR, leading to downstream signaling regulating proliferation of vascular smooth muscle cells [94]. 

Interestingly, also FPRs can stimulate proliferation in various types of cancer [40].

A role for uPAR in cell survival has also been proposed. uPAR, stimulated by endogenous uPA, prevents apoptosis of breast cancer cells by sustaining increased levels of activated ERK1/2 [95]. uPA-uPAR axis also promotes survival of retinal epithelial cells by activating Bcl-xL transcription [96]. Further, in glioblastoma cells, uPA suppresses the expression of BIM, a member of the pro-apoptotic BCL2-family protein; BIM suppression can be reversed by uPA silencing [97]. uPAR plays an important role in the survival of a neuroblastoma cell line through a mechanism probably involving the EGFR and its downstream effectors, since uPAR blocking results in the impairment of EGFR to activate signals for survival of mouse neuroblastoma cells [98]. 

uPAR may protect cells from apoptosis also indirectly. In fact, uPAR may up-regulate c-myc, which, in turn, enhances the expression of miR-17-5p and miR-20a; these miRs inhibit apoptosis by suppressing the expression of the death receptors 4 and 5 (DR4/DR5) [99].

### 3.5. uPAR in Cancer Metabolism

The energy metabolism in cancer cells is strongly dependent on aerobic glycolysis rather than on mitochondrial oxidative phosphorylation, leading to the preferential conversion of glucose to lactic acid, the so-called Warburg effect. Accordingly, the glucose uptake is strongly increased in cancer cells, since aerobic glycolysis is less efficient in terms of energy production as compared to mitochondrial oxidative phosphorylation [100]. 

Glioma cancer cells are able to shift from mitochondrial oxidative phosphorylation to aerobic glycolysis independently of oxygen availability. uPAR/MMP-9 silencing switches the glycolytic metabolism of glioma cells to oxidative phosphorylation (OXPHOS) and generates reactive oxygen species (ROS) to predispose glioma cells to mitochondrial outer membrane permeabilization. MMP-9/uPAR silencing activates complexes of mitochondria involved in OXPHOS and inhibits glycolytic hexokinase expression [101]. The uPA-uPAR axis is involved also in the aerobic glycolysis of melanoma cells. Indeed, silencing of uPAR expression or inhibition of uPAR interaction with integrins induces the inhibition of the PI3K/AKT/mTOR/HIF1 pathway, leading to impaired glucose uptake, decreased expression of various glycolytic enzymes and of PKM2, a checkpoint that controls metabolism of cancer cells; similar effects are also observed by silencing EGFR expression, suggesting its involvement in this uPAR activity [102]. More recently, uPAR knockout by (CRISPR)/Cas9 approach has been shown to induce a glycolytic and OXPHOS reprogramming in melanoma and colon carcinoma cell lines. In particular, in uPAR KO cells, authors observe an increased number of mitochondria in two melanoma cell lines and an immature biogenesis of mitochondria in the colon carcinoma line, accompanied by a significant enhancement of the mitochondrial respiratory capacity and a decreased glycolysis, even though with an increased secretion of lactate [103].

## 4. The Clinical Value of uPAR: Emerging Opportunities and Current Challenges in Cancer 

The clinical significance of uPAR in cancer arises from the important evidence that: (i) The expression of uPAR is increased in tumor tissues in respect to healthy tissues; (ii) the signaling pathways triggered by uPAR help cancer cells to escape from the cytotoxic effect of anti-cancer drugs. These observations have rendered uPAR a promising diagnostic and prognostic marker and an attractive target for clinical applications. 

### 4.1. Diagnostic Potential of uPAR in Malignancy

The diagnostic value of in vivo uPAR expression has been mainly analyzed by targeted radiopharmaceuticals or nuclear imaging [104,105]. Rabbani et al. were among the first to report the successful development of an antibody-based imaging probe directed to uPAR in order to exploit its diagnostic potential. The authors showed that the injection of a ^125^I-labeled polyclonal antibody directed to the ligand-binding NH_2_-terminal domain of uPAR is able to detect in vivo the presence of microscopic occult tumor metastases [106]. Subsequently, Dullin et al. showed that the administration of a Cy5.5-labeled uPAR-specific monoclonal antibody allowed the visualization of mammary carcinomas in an orthotopic mouse model, with high tumor specificity [107]. 

Peptide-based imaging probes against uPAR, as alternative approach to monoclonal antibodies, have been applied in several experimental studies, showing promising results for diagnostic PET imaging. To date, the studies on uPAR-targeted imaging are mainly focused on the 9-mer core peptide AE105, developed by combinatorial chemistry and characterized by high-affinity binding to human uPAR [108]. Li et al. provided the first demonstration of the ability of the AE105 peptide to visualize, by microPET imaging, uPAR expression in xenotransplanted tumor in mice. In this study, AE105 was conjugated with 1,4,7,10-tetraazadodecane-*N*,*N*′,*N*″,*N*‴-tetraacetic acid (DOTA) and labeled with ^64^Cu (^64^Cu-DOTA-AE105) [109]. Starting from these experimental settings, several strategies have been developed to improve the chemical characteristics and the pharmacokinetics of AE105 peptide and its derivatives, AE120 and AE170 [105]. 

In addition, quantitation of suPAR, the soluble form of uPAR, detectable in blood, saliva, plasma, serum, urine, ovarian cystic fluid and cerebrospinal fluid, represents an approach for diagnostic purposes [110]. 

suPAR was measured and evaluated for its diagnostic role in hepatocellular carcinoma (HCC). The early detection of high levels of suPAR in serum from patients with chronic liver disorders represents a specific and negative predictive marker in HCC [111]. In patients with hematologic malignancies, elevated serum suPAR level predicted infections in the early stage of febrile neutropenia with high sensitivity and negative predictive value [112]. In a study on correlation between inflammatory biomarkers and cancer, high plasma suPAR levels exhibited significant and independent value for cancer diagnosis in patients with nonspecific symptoms and signs of cancer [113]. A more recent article suggested that plasma suPAR measurement may help to distinguish between chronic pancreatitis and pancreatic cancer [114].

### 4.2. Prognostic Potential of uPAR in Malignancy

The clinical significance of uPAR as prognostic biomarker is associated with aggressive tumors and poor clinical outcome. The determination of uPAR has been performed in the different types of samples in which uPAR is detectable: biopsies for the evaluation of the plasma membrane uPAR; analysis of blood, plasma, and urine for suPAR. In particular, the shedding of suPAR into the plasma is currently considered a promising strategy to correlate the uPAR expression with aggressive tumor phenotype and/or with outcome of cancer. 

An emblematic example of the prognostic value of uPAR is its determination, by different approaches, in HCC. Several years ago, De Petro et al. found high uPAR mRNA levels by RT-PCR in HCC compared with those expressed in peritumoral hepatic tissues [115]. Dubuisson et al., by in situ hybridization, immunohistochemistry, and double immunofluorescence, established that uPAR transcripts and proteins were expressed mainly by stromal cells in HCC and were indicative of tumor progression and metastasis [116]. 

In patients with colorectal cancer (CRC), the preoperative concentrations of suPAR, analyzed by ELISA assay in plasma samples, were indicative of poor survival [117]. Afterwards, Loosen et al. demonstrated that serum levels of suPAR predicted the outcome after resection of colorectal metastasis [118].

Sier et al. performed suPAR measurement both on serum and urine samples of ovarian cancer patients in two independent studies. First, the authors showed that elevated serum suPAR levels in pre-operative but not post-operative patients were an indicator of poor survival prospects [119]. Then, they showed that urinary suPAR levels were enhanced in ovarian carcinoma patients and confirmed the enhanced serum and urinary suPAR levels derived from tumor tissues [120]. Later, Lyuca et al. proposed the monitoring of therapeutic successfulness of Platinum/Taxol-based chemotherapy by using serum suPAR determination in patients with ovarian carcinoma FIGO II [121]. 

A large body of evidence suggests the importance of uPAR as surrogate marker of aggressiveness in breast cancer. The prognostic value of uPAR in breast cancer was initially demonstrated [122]. Afterwards, it has been shown that high suPAR levels in pre-operative patients were associated with poor outcome in breast cancer independent of lymph node status, tumor size, and estrogen receptor status [123]. Moreover, RT-PCR analysis on micrometastatic cells isolated from breast cancer patients highlighted the correlation between elevated uPAR expression and poor prognosis during advanced stages of breast cancer [124].

Shariat et al. correlated elevated levels of circulating plasma suPAR with features of biologically aggressive prostate cancer, disease progression after prostatectomy, and metastasis [125]. However, further studies on prostate cancer showed that uPAR expression was not associated with adverse pathologic features or aggressive disease recurrence, thus the prognostic value of suPAR is still controversial [126]. 

The prognostic value of uPAR has been also characterized in lung carcinoma. ELISA analysis of tumor extracts from 84 patients with squamous cell lung carcinoma demonstrated that uPAR is an independent prognostic variable in squamous cell carcinoma patients [127]. Almasi et al., after developing time-resolved fluoroimmunoassay (TR-FIA) for uPAR, showed that DI-uPAR levels in the extracts of primary tumors predicted overall survival of 63 patients with squamous cell carcinoma (SCC) of the lung [128]. Prognostic implications of uPAR in patients with non-small-cell lung cancer (NSCLC) were suggested by Werle et al. In this report, uPAR levels were measured in homogenates of lung tumor tissue and of corresponding non-malignant lung parenchyma by ELISA assay. The data showed that uPAR is a prognostic factor for overall survival of NSCLC patients and is able to provide independent prognostic information on clinical and histological factors [129]. In a recent study, no associations were found between uPAR and poor prognosis [130], suggesting that further studies are needed to clarify the prognostic potential of uPAR in lung cancer patients. However, it has been recently demonstrated, in patient-derived tissue samples of NSCLC and colorectal cancer (CRC), that RAS mutational condition, a common mechanism of intrinsic resistance to EGFR inhibitors, is correlated to uPAR overexpression [131].

The clinical utility of suPAR has been also reported in blood cancers. Total suPAR levels increased in plasma from patients affected by acute myeloid leukemia and correlated with the number of circulating tumor cells. Notably, analysis of plasma, lysates of leukemic cells and urine samples from AML patients showed that DI-suPAR was only identified in urine. In patients undergoing chemotherapy, plasma DII-DIII-suPAR levels and urinary DI-suPAR levels decreased [132]. This result indicated that uPAR forms could be used for therapy monitoring. The prognostic significance of suPAR in AML has been later confirmed by Erkut et al., by analyzing serum suPAR levels by ELISA. The authors found positive correlation between suPAR levels and white blood cell counts. The median overall survival was longer in patients with serum suPAR levels below 6.71 ng/mL with respect to serum suPAR levels above 6.71 ng/mL [133]. 

### 4.3. uPAR as Target

The numerous observations about the central role of uPAR in tumor promotion and progression have provided the rationale for developing uPAR-targeted therapy.

Several uPAR inhibitors have been and are currently developed for suppression of tumor growth, metastatic processes and drug resistance. The various approaches include antagonist peptides, selective inhibitors of uPAR activities, monoclonal antibodies and gene therapy methods. Some of these approaches, such as peptides, small molecules, and antibodies, have been discussed in a previous review [134] and; therefore, summarized briefly here, in order to illustrate mainly the new evidences and technologies.

#### 4.3.1. Peptides- and Small Molecules-Derived Antagonists of uPAR 

One recently described approach is the design of peptides that antagonize uPAR interactions with uPA and with lateral membrane partners. Since several studies underlined the central role of the uPA/uPAR interaction in the invasion and metastasis processes of cancer cells, linear and cyclic peptides antagonists of uPA binding to uPAR were first produced. Through combinatorial chemistry approach, Ploug et al. identified a nonnatural 9-mer linear peptide antagonist of the uPA/uPAR interaction. This peptide was biologically active and inhibited intravasation of human cancer cells in a chick chorioallantoic membrane model [108]. Tarighi et al. investigated the effects of a decapeptide designed to impair the interaction of uPAR with its ligand. The uPAR antagonist decapeptide exerted pro-apoptotic effects on MDA-MB-231 breast cancer cells through down-regulation of Bcl-2 and up-regulation of Bim without Bax modulation [135].

The studies conducted by Carriero’s group led to the development of peptide-derived antagonists of uPAR, targeting the chemotactic ^88^Ser-Arg-Ser-Arg-Tyr^92^ sequence included in the flexible linker connecting DI and DII domains. uPAR antagonist pentapeptides carrying specific amino acid substitutions were developed and the pERERY-NH_2_ peptide was found to inhibit migration of cancer cells by blocking uPAR-FPRs interaction [136]. By an approach based on the conformational analysis of the ^88^Ser-Arg-Ser-Arg-Tyr^92^ sequence, RERF peptide, with a turned structure in solution, was indicated to prevent malignant cell invasion in vivo [137]. Interestingly, RERF exerted anti-angiogenic properties by blocking in vivo and in vitro responses triggered by uPAR_88-92_ or VEGF [138]. To optimize the biochemical properties for therapeutic applications, new antagonist peptides containing α-methyl α-amino acids were designed; among them, UPARANT was proposed as promising anti-cancer agent for its chemical properties and biological behavior in animal models [139]. In a recent study, cyclization of the SRSRY peptide exerted an anti-metastatic effect by reducing vascular infiltration by chondrosarcoma cells [140].

Another methodological approach to interfere with uPAR interactome is represented by small molecules drugs. IPR-456 and its derivative IPR-803 are small molecules inhibiting uPA/uPAR interaction identified by a virtual screening approach. These compounds show consistent effects on cell invasion of MDA-MB-231 breast cancer cells [141]. Notably, the orally administration of IPR-803 in female mice, inoculated with highly malignant TMD-MDA-MB-231 cells in their mammary fat pads, impaired metastasis formation to the lungs [142]. More recently, Meroueh’s group conducted a study of drug design and synthesis from which emerged small molecules compounds that bind to uPAR outside of the uPA/uPAR interface and block the receptor into a conformation that is not able to bind to uPA [143]. The same working group performed biophysical competition studies using uPAR mutants in order to develop more potent inhibitors of the uPA/uPAR interaction through small molecules strategy. The tested compounds were designed and synthesized employing the crystal structure of uPAR obtained from the uPAR/uPA complex [144]. Although these compounds have not yet been tested for their effects on cancer cells through in vitro and in vivo studies, they can lead to identification of new promising compounds for the development of anti-cancer drugs.

We used the virtual screening method to select small molecules targeting the uPAR-binding site for VN; we identified two compounds, C6 and C37, directed to S88 and R91, key residues in uPAR binding to VN as well as uPAR interaction with FPRs. In vitro and in vivo studies showed that C6 and C37 may be effective in preventing the metastatic process [131,145]. 

#### 4.3.2. Antibody-Based uPAR Inhibitors 

A panel of antibodies against various epitopes of uPAR has been developed and tested in vitro and in vivo. Positive results were obtained with the ATN-658 monoclonal antibody directed to the DIII domain of uPAR, able to inhibit cancer cells proliferation and survival. Evidences have been reported to support the advancing of the humanized version of ATN-658 (huATN-658) into phase 1 clinical evaluation [146].

The SRSRY sequence of the DI-DII linker region may represent a significant and innovative target for the development of new monoclonal antibodies. Indeed, we analyzed the effects of a polyclonal antibody directed to the SRSRY sequence; this antibody was able to inhibit uPAR-FPR interaction, CXCR4 activity and uPA- and fMLF-mediated cell adhesion and migration [47,50,59,134].

#### 4.3.3. uPAR-Targeting in Nanotechnologies

There is a growing interest for nanoparticles able to deliver drugs at the tumor site. Since uPAR expression is up-regulated in most cancer cells, uPAR-targeting nanoparticles may represent a useful tool. Wang et al. designed for the first time uPAR-targeting nanoparticles for prostate cancer cells; in this study, a GFD-derived peptide inserted into a liposome membrane was used to deliver plasmid DNA to uPAR-expressing cancer cells [147]. To allow the selective delivery to prostate cancer cells of noscapine, a tubulin-binding anti-cancer agent, uPAR-targeting optical-MR imaging trackable nanoparticles have been realized [148]. This approach may offer a great potential for prostate cancer patients. Yang et al. proposed the conjugation of iron oxide (IO) nanoparticles to ATF for delivery of ATF-IO to uPAR-positive breast cancer cells, as they bind to tumor cells in vitro and localize in tumor site in vivo [149]. Conjugation of a uPAR specific targeting peptide onto polyethylene glycol (PEG)-coated ultra-small super-paramagnetic iron oxide nanoparticles can potentially provide a useful supplement for tumor patient management, as it has been demonstrated by Hansen et al. [150]. These results are encouraging in further developing new anti-cancer strategies targeting uPAR.

#### 4.3.4. uPAR as Gene-Therapy Target: From Antisense Methodologies to Novel Gene-Editing Technologies

uPAR supports most of cancer cells activities; therefore, gene-based interference in its expression may represent a potential approach for cancer treatment. 

Antisense RNA technology has been first suggested by Go et al., which showed that human glioblastoma cells transfected with a construct corresponding to 300 bp of the human uPAR 5’ end in an antisense orientation, failed to invade fetal rat brain aggregates in vitro; further, stable transfectants, negative for uPAR expression, injected intracerebrally, failed to form tumors in nude mice [151]. Subsequently, Gondi et al. supported these data demonstrating that the intracranial injection of glioblastoma cells infected with a bicistronic construct containing antisense uPAR in an adenoviral vector impaired tumorigenicity and promoted disease regression in the established tumor [152]. Also the ability of non-small cell lung cancer to invade surrounding tissues and metastasize was inhibited by an uPAR antisense construct [153]. More recently, uPAR down-regulation with antisense oligodeoxynucleotide exerted an inhibitory effect on the invasive property of melanoma cells [68].

Small interfering RNAs (siRNAs) targeting uPAR expression have been proposed for the development of novel anti-cancer agents. Various approaches have been analyzed for in vivo delivery of siRNAs into tumors, but siRNA stability in blood and low efficiency delivery into tumor cells are the obstacles for application into anti-cancer therapy. However, in vivo promising studies on malignant meningiomas and then on glioma showed that uPAR down-regulation by siRNA technology induced tumor regression [154,155].

Another possible strategy to target uPAR expression may be the use of small non-coding RNAs (miRs). We identified oncosuppressor miRs, in particular miR-146a and miR-335, targeting uPAR mRNA in acute myeloid leukemia cell lines and blasts (52). Indeed, miR-146a may represent a useful tool for the development of therapeutics since its deletion in mouse models leads to myeloproliferative disorders. Wach et al. have recently established the therapeutic relevance of miR-143, that targets uPAR in prostate cancer, playing a major role against tumor cell dissemination and metastasis [156]. 

Recent advances in genome engineering technologies based on the CRISPR-associated RNA-guided endonuclease Cas9 (CRISPR/Cas9) is a versatile gene-editing technology to modify, delete or correct precise regions of genome. Wang et al. recently demonstrated that targeting uPAR by CRISPR/Cas9-based genome editing in colon carcinoma HCT8 and multiple-drug resistant KB_V200_ cell lines resulted in attenuation of malignant phenotype and multidrug resistance [157]. Although there are several limitations in the clinical application of CRISPR/Cas9 system, it is conceivable that cancer patients will benefit of this technology in the near future. 

The CAR T-cell therapy represents a new era in cancer immunotherapy. Wang et al. produced the first study that reports uPAR as a candidate target for CAR T-cells therapy in ovarian cancer. Anti-uPAR chimeric antigen receptors were designed using ATF, that is the natural ligand of uPAR. ATF CAR T-cells exhibited effective anti-cancer activity against uPAR-positive ovarian cancer cells [158]. 

The strategy of anti-uPAR CAR T-cells was applied also to treatment of senescence-associated disorders. uPAR-specific CAR T-cells extend the survival of mice with lung adenocarcinoma treated with senescence-inducing drugs and revert liver fibrosis phenotype [84]. Taken together, these data suggest that CAR T-cell could be an excellent strategy for the future treatment of uPAR-positive cancers.

**Figure 1:**
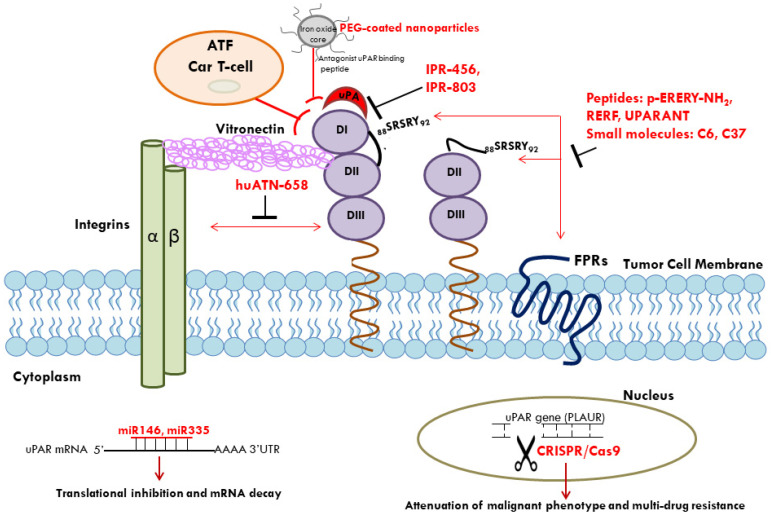
Multiple approaches for uPAR-targeted therapy in cancer. uPAR-targeted therapy includes antagonist peptides, small molecules, monoclonal antibodies, and gene-therapy techniques for suppression of tumor growth, metastasis, and drug resistance. uPA/uPAR interaction can be inhibited by small molecules such as IPR-456 and its derivative IPR-803. The SRSRY sequence, able to interact with FPRs and to mediate cell chemotaxis, represents a target for peptides (p-ERERY-NH2, RERF, UPARANT) and small molecules (C6, C37). huATN-658 monoclonal antibody, directed to DIII domain of uPAR, inhibits uPAR/Integrin association. Conjugation of a specific uPAR-targeting antagonist peptide to polyethylene glycol (PEG)-coated iron oxide nanoparticles can potentially provide a useful supplement for tumor patient management. Oncosuppressor miRs, such as miR-146a and miR-335, targeting uPAR mRNA, may represent useful tools for the development of anti-cancer therapy. uPAR-targeting by CRISPR/Cas9-based genome editing results in attenuation of malignant phenotype and multidrug resistance. Finally, uPAR may represent an appropriate target for CAR T-cells; anti-uPAR chimeric antigen receptors were designed using the amino-terminal fragment (ATF) of uPA; ATF CAR T-cells exhibit anti-cancer activity against uPAR-positive ovarian cancer cells. uPAR: urokinase receptor; FPRs: fMLF receptors; miRs: microRNAs.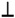: inhibition.

In conclusion, uPAR may represent a useful marker in diagnosis and prognosis of various cancers; up-regulation of uPAR expression in cancers cells makes this receptor an attractive target also in innovative anti-cancer therapeutic strategies (Figure 1).

## Data Availability

Not applicable.
